# Prognostic factors of poor postoperative outcomes in gastrectomies

**DOI:** 10.3389/fsurg.2023.1324247

**Published:** 2023-12-01

**Authors:** B. O. Stüben, G. A. Plitzko, L. Stern, J. Li, J. P. Neuhaus, J. W. Treckmann, R. Schmeding, F. H. Saner, D. P. Hoyer

**Affiliations:** ^1^ Department of General, Visceral and Transplant Surgery, Medical Centre University Duisburg-Essen, Essen, Germany; ^2^ Department of General, Visceral and Thoracic Surgery, University Medical Center Hamburg-Eppendorf, Hamburg, Germany; ^3^ Department of Surgery, Jiahui International Hospital, Shanghai, China; ^4^ Organ Transplant Center of Excellence, King Faisal Specialist Hospital & Research Center, Riyadh, Saudi Arabia

**Keywords:** gastrectomy, mortality, morbidity, emergency surgery, subtotal gastrectomy

## Abstract

**Background:**

Gastric cancer is one of the most common cancers worldwide and is the third most common cause of cancer related death. Improving postoperative results by understanding risk factors which impact outcomes is important. The current study aimed to compare immediate perioperative outcomes following gastrectomy.

**Methods:**

302 patients following gastric resections over a 10-year period (January 2009–January 2020) were identified in a database and retrospectively analysed. Epidemiological as well as perioperative data was analysed, and a univariate and multivariate analysis performed to identify risk factors for in-hospital mortality.

**Results:**

In general, gastrectomies were mainly performed electively (total vs. subtotal 95% vs. 85%, *p* = 0.004). Patients having subtotal gastrectomy needed significantly more PRBC transfusions compared to total gastrectomy (*p* = 0.039). Most emergency surgeries were performed for benign diseases, such as ulcer perforations or bleeding and gastric ischaemia. Only emergency surgery was significantly associated with poorer overall survival (HR 2.68, 95% CI 1.32–5.05, *p* = 0.003).

**Conclusion:**

In-hospital mortality was comparable between total and subtotal gastrectomies. Only emergency interventions increased postoperative fatality risk.

## Introduction

Gastric resection remains the preferred surgical treatment option for many malign and benign diseases of the stomach.

Currently, the most common indication for a gastric resection is gastric cancer, while only seldomly performed for peptic ulcer disease. Gastric cancer is one of the leading causes of cancer related deaths and almost two-thirds of cases occur in developing countries (1, 2). Gastric resection remains the only curative therapy, with the development of multimodal concepts leading to significantly improved oncological outcomes in patients with locally advanced gastric cancer (3).

The extent of gastrectomy for curative treatment of gastric cancer depends on tumour location, tumour size and tumour stage (4, 5).

Perioperative outcomes have improved decidedly in the last years, with perioperative mortality of 15% in the 1990's being reduced to under 5% in more recent years (6, 7). However, this mortality rate is still higher than that described for both pancreaticoduodenectomy and major hepatectomy (8, 9). Additionally, postoperative complication rates are high ranging from 9%–46% following total gastrectomy (10, 11). These complications have an impact on length of hospital stay, in hospital and overall mortality and recurrence rates (12, 13). The relationship between complications and long-term outcomes have not yet been fully understood, however it has been shown that patients with postoperative complications are more likely to not receive adjuvant therapy following surgery (10, 14). In Europe, there have been efforts in recent years to standardise the reporting of complications following gastrectomy for cancer, leading to the formation of the GASTRODATA registry (15).

Data from European and Asian studies allow for the consensus that for locally advanced tumours, subtotal gastrectomy can substitute for total gastrectomy provided there are sufficient tumour-free resection margins (4, 16–19). While resection margins are important for ensuring R0 resections, the exact length of margin required is a matter of controversy. Historically, safety margins of 8 cm for diffuse type and 5 cm for intestinal type carcinomas have been the accepted standard. For intestinal types, this was confirmed by a study in the United States, where margins of 3–5 cm demonstrated a prognostic advantage, while margins over 5 cm did not (20).

Apart from gastric cancers, there are many other malignant diseases which require gastric resection, including gastrointestinal stroma tumours, neuroendocrine tumours, sarcomas and sometimes lymphomas. Peptic ulcers must also be surgically treated if they lead to endoscopically uncontrollable bleeding or free perforation.

There is conflicting evidence regarding the morbidity of subtotal gastrectomy compared to total gastrectomy. While one study showed that subtotal gastrectomy led to a reduction in anastomotic leaks without an effect on overall morbidity compared to total gastrectomy, two meta-analyses showed a decrease in overall postoperative complications after subtotal gastrectomy with equivalent long term oncological results (21–23).

The current study aims to assess differences of in-hospital outcomes between subtotal and total gastrectomy and investigate whether certain risk factors predispose for worse short-term outcomes following gastric surgery.

## Methods

### Data collection

302 patients receiving a gastric resection at the Department of General, Visceral and Transplant Surgery, Medical Centre University Duisburg-Essen, Germany (admitted from January 2008 to January 2020) were identified in a database and retrospectively analysed.

### Patient data, exclusion criteria

We analysed age, sex, and body mass index (BMI) from our electronic hospital database. Tumour size, tumour number, tumour differentiation, vascular invasion, and Union for International Cancer Control (UICC) stage were determined postoperatively by the pathologist for patients with malignant disease. Patients with distant metastasis or peritoneal seeding as well as those lost to follow up were excluded from the study. Patients who had partial or wedge non-anatomic gastric resection and did not require gastrointestinal reconstruction were not included in this study.

### Choice of surgery

The decision on which resection and reconstruction technique was performed was made intraoperatively by an experienced upper GI surgeon. Indication for resection, defined by final post-operative diagnosis including pathologic confirmation, was stratified into malignant and benign diagnoses using the International Classification of Diseases (ICD) 11th revision.

### Outcome and follow-up

All complications available in our database were chosen and analysed according to the NSQIP database (24). These included pulmonary, medical, cardiovascular, and surgical complication rates as well as the amount of blood transfusion required intraoperatively. The primary outcome of the study was the in-hospital mortality rate.

Secondary outcomes were overall complication and survival rates.

### Ethics approval

In accordance with German law, approval by a local ethics committee was not required (paragraph 15, sentence 1, North Rhine Medical Association's professional code of conduct from 14 November 1998 as amended on 19 November 2011), and written informed consent was not obtained from the participants because of the strict retrospective design of our study (paragraph 6, sentence 1, Health Data Protection Act of North Rhine-Westphalia).

### Statistical analysis

All statistical tests were performed using R Statistical software (version 4.2.1; R Foundation for Statistical Computing, Vienna, Austria) and Graph Pad Prism Software (version 9.2.0; GraphPad Software Inc., La Jolla, CA, USA). Quantitative non-normally distributed variables were stated as median ± interquartile range (IQR). Percentages are based on the respective subgroups as indicated. The quantitative and qualitative variables’ association was assessed using the Wilcoxon-rank test for non-normal distributions. The relationship between qualitative variables was tested with Chi-Square or Fisher's exact test when the sample size was <5. To assess independent predictors of in-hospital mortality following gastric surgery a multivariable analysis was performed using a logistic regression model. Only parameters with statistically significant relationships in the univariate analysis were introduced into the multivariable model to detect parameters independently associated with major postoperative complications. The Hosmer–Lemeshow test was used to evaluate the goodness of fit of the logistic model. The results were presented as odds ratios with 95% CI.

All tests were two-tailed at a significance level of <0.05.

## Results

### Baseline demographics and treatment data

A total of 302 patients received a gastric resection from January 2008 to January 2020 and were included in the final analysis after application of the exclusion criteria.

The median (IQR) age was 62 (57–72) years.

There was a male predominance across the cohort (58% vs. 42%). Median (IQR) BMI was 25 (22.7–28.7) kg/m^2^.

The most common comorbidity was diabetes, with 38 (13%) patients affected. This was followed by coronary heart disease (CHD) in 31 (11%) patients. Alcohol and nicotine abuse was seen in 10 (4%) and 50 (17%) patients respectively. 25 patients (8.6%) patients had chronic kidney disease of varying stages.

79 (39%) patients had American Society of Anaesthesiologists (ASA) I–II classification, with 122 (51%) being ≥ASA III.

138 (46%) patients received subtotal or proximal gastrectomy, with 164 (54%) receiving a total gastrectomy. For reconstruction, Roux-Y was the most common with 78%, followed by Billroth I and II reconstructions (8% and 11% respectively). A multivisceral resection was necessary in 35% of cases, as defined by resection of at least one neighbouring organ. 87% of patients were treated for a malignant disease, with adenocarcinomas being the most common malignancy with 62%. When looking at UICC staging, there were no significant differences between subtotal gastrectomy and total gastrectomy.

Of the patients with malignant disease, 148 (51%) received neoadjuvant chemotherapy (CTx) and 5 (2%) neoadjuvant radiotherapy (RTx).

91% of patients received elective surgery, with significantly more total gastrectomies performed electively than subtotal gastrectomies (95% vs. 85%, *p* = 0.004).

The majority (70%) of patients received a D2 lymphadenectomy, with 20% receiving no lymphadenectomy since these were treated for benign disease. There was a significant difference in the extent of lymphadenectomy performed, with significantly more patients receiving a gastrectomy also having an extensive lymphadenectomy (D2 or D3) than patients with subtotal gastrectomy (83% and 5% vs. 54% and 0%, *p* < 0.001).

There was significantly more need for the transfusion of packed red blood cells (PRBC) in the subtotal gastrectomy group compared to total gastrectomy (20 vs. 14, *p* = 0.039).

The baseline demographics as well as treatment data are presented in Table 1.

**Table 1:** Baseline and treatment data.

Subgroup	Data available	Overall (*n* = 302)	Proximal/subtotal gastrectomy (*n* = 138)	Total gastrectomy (*n* = 164)	*p*-value
Baseline					
Age, yrs	302	62 (52, 72)	63 (52, 72)	61 (53, 71)	0.4
Male	302	175 (58%)	79 (57%)	96 (59%)	0.8
BMI, kg/m²	302	25.0 (22.7, 28.7)	24.9 (22.9, 28.7)	25.2 (22.6, 29.0)	0.8
ASA	201				
I–II		79 (39%)	33 (38%)	46 (41%)	0.6
≥III		122 (51%)	55 (62%)	67 (59%)	
Diagnosis	** **				**<0**.**001**
Adenocarcinoma	302	188 (62%)	52 (38%)	136 (83%)	
NET		23 (8%)	15 (11%)	8 (4.9%)	
GIST		24 (8%)	19 (14%)	15 (9%)	
other		67 (22%)	52 (38%)	15 (9%)	
Malignancy	** **	257 (87%)	106 (77%)	151 (94%)	**<0**.**001**
Tumour stage					
UICC I		54 (26%)	23 (34%)	31 (22%)	0.07
UICC II–IV		151 (74%)	44 (66%)	107 (78%)	
Comorbidities					
CKD	292				0.4
1		1 (<1%)	1 (<1%)	0 (0%)	
2		6 (2%)	3 (2.3%)	3 (2%)	
3		12 (4%)	5 (3.8%)	7 (4%)	
4		3 (1.0%)	3 (2.3%)	0 (0%)	
5		3 (1%)	2 (2%)	1 (<1%)	
none		267 (91%)	119 (89%)	148 (93%)	
Diabetes	288	38 (13%)	20 (15%)	18 (11%)	0.3
CHD	288	31 (11%)	20 (15%)	11 (7%)	**0**.**022**
Alcohol	288	10 (4%)	4 (3.1%)	6 (3.8%)	>0.9
Tobacco	288	50 (17%)	24 (18%)	26 (16%)	0.7
neoadjuvant CTx	288	148 (51%)	46 (35%)	102 (65%)	<0.001
neoadjuvant RTx	288	5 (2%)	1 (0.8%)	4 (3%)	0.4
Intraoperative					
Transfusion RBC no.	203	34 (17%)	20 (23%)	14 (12%)	**0**.**039**
Lymphadenectomy	284				**<0**.**001**
D1		24 (9%)	17 (13%)	7 (5%)	
D2		200 (70%)	69 (54%)	131 (83%)	
D3		7 (3%)	0 (0%)	7 (5%)	
none		53 (19%)	41 (32%)	12 (8%)	
Reconstruction	282				**<0**.**001**
B1		23 (8%)	23 (19%)	0 (0%)	
B2		32 (11%)	29 (24%)	3 (2%)	
Roux-Y		221 (78%)	68 (56%)	153 (96%)	
Longmire		6 (2%)	2 (1.6%)	4 (3%)	
Multivisceral	290	101 (35%)	52 (39%)	49 (31%)	0.2
Elective surgery	299	271 (91%)	116 (85%)	155 (95%)	**0**.**004**

BMI, body mass index; ASA, American Society of Anaesthesiologist's classification; NET, neuroendocrine tumour; GIST, gastrointestinal stromal tumour; UICC, union for international cancer control; CKD, chronic kidney disease; CHD, coronary heart disease; CTx, chemotherapy; RTx, radiotherapy; B1, Billroth 1; B2, Billroth 2.

Data presented as median (IQR) or presented as *n* (%).

Statistical significance was tested using Chi-Square or Fisher's exact test when the sample size was <5 for qualitative variables and with the Mann–Whitney test for continuous variables with non-normal distributions. Bold *p*-values are of statistical significance (≤0.05).

### Complications

#### Pulmonary

18 (6%) patients developed pneumonia. A total of 18 (6%) patients developed an ARDS following surgery, with 8 (44%) secondary to postoperative pneumonia and 10 (56%) patients developing ARDS due to other reasons. The pneumonia and ARDS rates did not significantly differ between the subtotal and total gastrectomy groups.

#### Cardiovascular

4 (1%) patients developed a postoperative myocardial infarction (MI), while 2 (1%) patients developed pulmonary embolism (PE). All myocardial infarctions occurred in the subtotal gastrectomy group.

#### Medical

16 (6%) showed acute kidney injury (AKI) and a total of 11 (4%) required continuous veno-venous haemodialysis (CVVHD) in the postoperative course.

Sepsis occurred in 19 (7%) patients. There were no significant differences in the postoperative medical complication rates between the subtotal and total gastrectomy groups.

#### Surgical

Bleeding as defined by the need for the transfusion of at least one unit of PRBC postoperatively occurred in a total of 9 (3.2%) patients.

A surgical site infection (SSI) occurred in 27 (10%) patients.

23 (8%) had an anastomotic leak, with anastomotic stenosis (endoscopically confirmed and requiring at least one endoscopic intervention) occurring in 2 (1%) patients.

63 (25%) patients developed a major complication defined as Clavien–Dindo grade III and more.

The incidence of complications is shown in Table 2.

**Table 2:** Type of complications.

	Data available	Overall (*n* = 302)	Proximal/subtotal gastrectomy (*n* = 138)	Total gastrectomy (*n* = 164)	*p*-value
Pulmonary					
Pneumonia	285	18 (6%)	5 (4%)	13 (8%)	0.13
ARDS	285	18 (6%)	9 (7%)	9 (6%)	0.7
Cardiovascular					
MI	285	4 (1%)	4 (3%)	0 (0%)	**0**.**040**
PE	284	2 (1%)	1 (1%)	1 (1%)	>0.9
Medical					
AKI	284	16 (6%)	5 (4%)	11 (7%)	0.3
CVVHD	285	11 (4%)	7 (6%)	4 (3%)	0.2
Sepsis	285	19 (7%)	6 (5%)	13 (8%)	0.2
Surgical					
Bleeding	285	9 (3.2%)	4 (3%)	5 (3%)	>0.9
SSI	285	27 (10%)	11 (9%)	16 (10%)	0.6
Anastomotic leak	285	23 (8%)	9 (7%)	14 (1%)	0.6
Anastomotic stenosis	285	2 (1%)	1 (1%)	1 (1%)	>0.9
Clavien–Dindo	252				
0		39 (15%)	15 (13%)	24 (17%)	0.5
I–II		150 (60%)	67 (59%)	83 (60%)	
≥III		63 (25%)	32 (28%)	31 (22%)	

ARDS, acute respiratory distress syndrome; MI, myocardial infarction; PE, pulmonary embolism; AKI, acute kidney injury; CVVHD, continuous veno-venous haemodialysis; SSI, surgical site infection.

Data presented as *n* (%).

Statistical significance was tested using Chi-Square or Fisher's exact test when the sample size was <5 for qualitative variables and with the Mann–Whitney test for continuous variables with non-normal distributions. Bold *p*-values are of statistical significance (≤0.05).

#### Outcomes

The patient cohort had an in-hospital mortality rate of 10% (29 patients). There was no statistically significant difference in the in-hospital mortality rate between the subtotal and total gastrectomies.

The failure to rescue rate was 13.6%.

The overall median (IQR) length of stay (LOS) was 15 (11–21) days and did not differ significantly between subtotal and total gastrectomies.

The median (IQR) duration in the intensive care unit (ICU) was 1.1 (0.8–3.6) days, also without significant differences between the groups.

The in-hospital treatment outcomes are shown in Table 3.

**Table 3:** In-hospital outcome.

	Data available	Overall (*n* = 302)	Proximal/subtotal gastrectomy (*n* = 138)	Total gastrectomy (*n* = 164)	*p*-value
In hospital Mortality	301	29 (10%)	16 (12%)	13 (8%)	0.3
LOS, days	302	15 (11, 21)	14 (10, 23)	15 (12, 20)	0.3
ICU, days	302	1.1 (0.8, 3.6)	1.0 (0.8, 3.2)	1.7 (0.8, 3.9)	0.086

LOS, length of stay; ICU, intensive care unit.

Data presented as median (IQR) or presented as *n* (%).

Statistical significance was tested using Chi-Square or Fisher's exact test when the sample size was <5 for qualitative variables and with the Mann–Whitney test for continuous variables with non-normal distributions.

To investigate whether in-hospital mortality was indeed the same for each procedure and thus not procedure-related, and to investigate for possible risk factors which may lead to a higher in-hospital mortality rate, we carried out a multivariate analysis where we tested all parameters which were significantly different between total and subtotal gastrectomy and which may influence mortality rates. These included CHD and malignant disease as comorbidities, and emergency surgery as procedure related. Here we were able to show that while non-malignant disease showed a trend towards higher in-hospital mortality, only emergency surgery was found to be a statistically significant risk factor of in-hospital mortality (HR 6.65, 95% CI 1.87–24.22, *p* = 0.003).

The results of the multivariate regression analysis are shown in Table 4.

**Table 4:** Multivariate regression of factors influencing in-hospital mortality following gastrectomy.

Variable	Hazard ratio [95% CI]	*p*-value
Procedure [total]	1.04 [0.41–2.69]	0.940
Surgery [emergency]	6.65 [1.87–24.22]	**0**.**003**
Malignancy [yes]	0.411 [0.12–1.5]	0.177
CHD [yes]	1.28 [0.19–5.09]	0.757

CHD, coronary heart disease. Statistical significance was tested using a multivariate logistic regression. Odds ratio for in-hospital mortality are reported with *p* values. Bold *p*-values are of statistical significance (≤0.05).

## Discussion

We found that no significant differences exist between total and subtotal gastrectomy when looking at in-hospital mortality.

We were able to demonstrate that only emergency surgery was significantly associated with poorer OS (HR 2.68, 95% CI 1.32–5.05, *p* = 0.003). This is in line with previously published studies which show that emergency abdominal surgery generally shows higher postoperative mortality rates (25, 26). Additionally, a study focusing on emergency gastric surgery found in-hospital mortality rates of up to 50% (27). Notably, 4 patients with complete gastric ischemia were among the emergency operations, all of which died in the perioperative course. The poor prognosis of patients with gastric ischemia has been well documented in prior studies (28, 29).

Patients receiving subtotal gastrectomy needed significantly more PRBC transfusions compared to total gastrectomy (*p* = 0.039). Most subtotal gastrectomies were performed for benign diseases in emergency situations, such as ulcer perforations or bleeding and gastric ischaemia in patients with reduced performance status. The combination of reduced performance status and emergency surgery could explain the higher rate of PRBC transfusions for subtotal gastrectomies. Another explanation for our findings that patients with total gastrectomy required less PBRC transfusions could be that in these surgeries, all gastric vessels are ideally controlled and excluded before resection, usually reducing intraoperative bleeding significantly.

When analysing overall complication rates, we saw no statistically significant differences in pulmonary, medical, and surgical complication rates following total or subtotal gastrectomy. Importantly, anastomotic complications (leak or stenosis) were both not significantly different between the two surgical procedures. This is contrary to two previous studies from Asia which investigated total versus subtotal gastrectomy in patients with gastric cancer, where one study described lower anastomotic insufficiency rates in patients receiving subtotal gastrectomy compared to total gastrectomy, with no effect on overall morbidity rates (21). A further study in fact showed lower overall complication rates in subtotal gastrectomy with equivalent long-term oncological results (22).

When looking at short-term outcomes, we were able to show that in-hospital mortality rates, LOS und ICU days were similar in both groups.

To date, there have been multiple studies published comparing total and subtotal gastrectomy, mostly for patients with gastric cancer. There are other malignant diseases which necessitate gastrectomy, including gastrointestinal stroma tumours, neuroendocrine tumours, sarcomas and lymphomas. Benign diseases which must be surgically treated are peptic ulcer disease leading to perforation or uncontrollable bleeding. A seldom but important indication is emphysematous gastritis, a rare disease with gastric inflammation and intramural gas formation due to gas-forming microorganisms, and in which rapid diagnosis and emergency gastrectomy are vital high, and which if left untreated shows high mortality rates (30).

Many surgeons now perform subtotal gastrectomy to preserve the gastric remnant, and data from Asian and European studies have shown that from an oncological standpoint, subtotal gastrectomy can be performed instead of total gastrectomy in locally advanced tumours (4, 16–19). However, both total and subtotal gastrectomy are associated with heavy morbidity and mortality, as shown by the GASTRODATA registry which analysed 1,349 gastrectomies for cancer over a one-year period across several European countries (15). Based on this registry, further studies since its creation have also shown that morbidity and mortality rates after gastrectomy remain high and present a real problem (31, 32).

Our study has certain limitations. It is a single-centre and low-volume study. Due to the retrospective nature of the study, the patient's postoperative nutritional status and proportional weight loss as well as information of quality-of-life following surgery is lacking.

We were only able to include the complications which were available in our retrospective database. While these include most major complications listed by the NSQIP manual, we have not been able to analyse all complications due to availability. While we have analysed a heterogeneous group in terms of disease spectrum, we only have a small number of benign conditions. This is however reflective of the case load in most tertiary medical centres regarding gastric resections. We were only able to include mortality as a parameter as the statistical power for single complications was low due to relatively low case numbers in each group. The statistical power of the data is limited by the relatively low case numbers; therefore, subgroup analyses were not performed.

## Conclusions

No significant differences exist between total and subtotal gastrectomy when looking at in-hospital mortality. Only emergency surgery was identified as a risk factor for higher postoperative mortality.

## Data Availability

The original contributions presented in the study are included in the article/Supplementary Material, further inquiries can be directed to the corresponding author.
